# Unmet support needs in teenage and young adult childhood brain tumour survivors and their caregivers: “it’s all the aftermath, and then you’re forgotten about”

**DOI:** 10.1007/s00520-021-06193-x

**Published:** 2021-04-16

**Authors:** Emma Nicklin, Lucy Pointon, Adam Glaser, Naseem Sarwar, Michelle Kwok-Williams, Miguel Debono, Galina Velikova, Florien W. Boele

**Affiliations:** 1 Leeds Institute of Medical Research at St James’s, University of Leeds, Leeds, UK; 2 Leeds Teaching Hospitals NHS Trust, Leeds, UK; 3 Sheffield Teaching Hospitals NHS Foundation Trust, Sheffield, UK; 4 Leeds Institute of Health Sciences, University of Leeds, Leeds, UK

**Keywords:** Brain tumour, Teenage young adult, Survivorship, Family caregiver, Supportive care, Unmet needs, Late effects

## Abstract

**Purpose:**

Teenage and young adult (TYA) survivors of childhood brain tumours and their family caregivers can experience many late effects of treatment that can hamper the transition to living independent lives. Yet, their long-term supportive care needs are largely unknown. We investigated the supportive care needs of TYA survivors and their caregivers and explored the role and perceived use of support.

**Methods:**

Face-to-face semi-structured interviews were conducted with survivors aged 16–30 (*n* = 11) who were ≥ 5 years after diagnosis and caregivers (*n* = 11). Interviews were recorded and transcriptions thematically analysed.

**Results:**

Four themes emerged: (1) *preferences for support and support services (unmet needs)*. Concerns regarding mental health, employment and financial uncertainty, the desire to live independently, and lack of support were emphasised. (2) *Decline in support*. Caregivers noted a drop-off in support available when transitioning to adult services. (3) *Reasons for not obtaining adequate support*. Several barriers to accessing support were raised, including distance and aging out of services. (4) *The role of long-term hospital-based follow-up care*. Participants highlighted the importance of, and reassurance from, long-term follow-up care but noted a more all-inclusive approach is required.

**Conclusions:**

Even many years after diagnosis, TYA childhood brain tumour survivors and their caregivers continue to have unmet supportive care needs. Both TYA survivors and their caregivers can benefit from support to meet their unique needs and improve long-term quality of life. Understanding unmet needs and recognising what services are required due to the late effects of treatment is critical to improving long-term quality of survival.

## Introduction

In children, there are over 100 histological entities for childhood brain tumours, the most common of which are gliomas accounting for around 50% [1, 2]. Incident rates for children aged 0–19 for central nervous system tumours are 6.06 per 100,000 [2]. Many children become teenage and young adult cancer survivors (TYAs — broadly defined in the literature as patients aged 13–39 [3]), facing specific challenges different from those of younger children or older adults.

Treatment chosen depends on tumour type, location, and age, often involving multimodal approaches including surgery, irradiation, chemotherapy, and medication [4]. While treatments for childhood cancer have improved in minimising adverse effects and maximising long-term survival [5, 6], survivors commonly experience substantial changes in their developmental trajectories [7]. With longer survival, late effects (e.g. physical, social, emotional, behavioural, and neurocognitive issues) are particularly important for quality of life (QoL). Indeed, childhood brain tumour survivors are at risk of worse QoL when compared to the general population and other TYAs [8–10].

A childhood brain tumour also impacts greatly on those closest to the patient. The responsibility for caring and supporting childhood brain tumour survivors is often met by their parents. Adapting to a new role as informal caregiver can be rewarding but also causes significant distress and burden [11]. With care demands combining those of cancer and a brain injury, high caregiver burden is associated with depressive symptoms and lower self-esteem [11–13]. Understanding caregivers’ and survivors’ ongoing support needs is vital.

TYAs remain at risk of late effects [14, 15]. TYAs frequently experience cognitive deficits, such as memory and attention difficulties which can have further negatively impact on cognitive development, independent living, academic achievement, and social functioning [16–18]. Many TYAs have difficulties in navigating social and romantic relationships [19, 20]. They are often faced with tension between their emerging abilities as they move through adolescence and into young adulthood and their limitations imposed by their illness [21].

These challenges can persist for the survivors’ lifetime and may require long-term tailored support and care. Long-term follow-up clinics can offer coordinated, multidisciplinary care addressing survivors’ and caregivers’ issues [22–24]. To improve services, having a clear overview of unmet supportive care needs of TYA survivors of childhood brain tumours and their caregivers is crucial — yet, we found little research addressing this in our recent systematic review [25]. We aimed to describe supportive care needs of TYA childhood brain tumour survivors and their caregivers and explore the role and perceived use of support services. More knowledge in this area is critical to improving quality of survival.

## Methods

### Study design

We present the qualitative work stream of a larger mixed methods project. The current project consists of in-depth, semi-structured face-to-face interviews. The consolidated criteria for reporting qualitative research (COREQ) were used in this report [26]. All quotes are pseudonymized. The study was approved by Yorkshire & the Humber — Bradford Leeds Research Ethics Committee (18/YH/0312).

### Participants

TYA childhood brain tumour survivors (hereafter called “survivors”) and their caregivers were recruited from long-term follow-up clinics at Leeds and Sheffield National Health Service (NHS) Trusts between November 2018 and January 2020, using maximum variation sampling to ensure a varied range of sex, age, and brain tumour type. The lead author (EN) liaised closely with clinicians (including AG, NS, MKW, MD) to ensure participants were representative of the population.

Survivors were invited to participate if aged between 13 and 30; diagnosed before the age of 14; ≥ 5 years post-treatment; and able to understand English. Caregivers were eligible to participate in the study if they were English-speaking, primary caregivers of a survivor. Survivors and caregivers did not have to be pairs from the same family. Written consent was obtained from all participants.

### Data collection

The interviews followed an interview guide (Table 1) consisting of open-ended questions, developed through a systematic review [25]. Participants were interviewed individually; however if preferred (e.g. if the survivor required support), survivors and caregivers could be interviewed together. Data collection ceased when saturation was achieved [27]. All interviews were audio recorded and transcribed verbatim.

**Table 1:** Interview guide

Interview topics	Key questions
Survivor — after diagnosis/treatment	- How has life been since [survivor’s] brain tumour diagnosis? -Changes/difficulties faced? -Have there been any impacts to employment or education?
Caregiver challenges	- What are the main changes/challenges since the diagnosis/treatment? - Have these challenges changed since [survivor] entered adolescence? If so how?
Support services and service use	**Survivor questions** - What services have been involved in your after care? (e.g. charities) - When did you receive this (during/after treatment)? - Could these services be improved? **Caregiver questions** - What support did you receive and how were you made aware? - Do you think these services could be improved?
Long-term follow-up care	- What is the benefit of attending clinics? - Is what is discussed in clinic is understandable for you? (**Survivor specific**) - Do you feel that these clinics are age appropriate for survivors? (**Caregiver specific**)
Support recommendations	- Is there anything you would have liked or would now like more support with? **Distinguish between patient support and caregiver support.** - What information should be given to survivors and caregivers? - Are there any particular resources that should be provided for survivors as they become older? - How can support and the way you are informed about support be improved?

### Analysis

Thematic analysis [28] was conducted by one coder (EN, PhD candidate), supported closely by more senior researchers (FWB and GV, supervisors). We used Braun and Clarke’s [29] 6-step framework, (step 1, transcription, immersion, and familiarisation; step 2, generating initial codes; step 3, searching for themes; step 4, reviewing and revising themes; step 5, defining themes; step 6, writing-up) to develop a clear and consistent approach to exploring survivors and their caregivers’ experiences. Following verbatim transcription [30], the transcripts were re-read to promote “immersion in the data”, allowing familiarisation to occur. The data could then be read actively, analytically, and critically [31]. Coding was conducted using NVivo software, with a subset of four interviews coded independently by a second coder (FWB), both coders then met to discuss and refine codes. Key outcomes were refined into themes reflecting participants’ experiences. Survivor and caregiver interviews were analysed simultaneously. Finally another researcher (LP, research assistant) examined all transcripts post-analysis to ensure analytical robustness and validity. Throughout analysis, any differences or disagreements between coders or checkers (EN, FWB, LP, GV) were resolved through discussion and/or re-examination of transcripts.

## Results

In total 21 survivors and 18 caregivers were approached, 22 (11 survivors; 11 caregivers) consented and took part. Interviews were conducted in participants’ home or a hospital room. In two cases, survivors and caregivers were interviewed together. Interviews averaged 53 minutes (range: 15–140). Data saturation was reached by the 22nd interview.

Tables 2 and 3 show participant characteristics. The most common tumour type was astrocytoma (survivor sample) and medulloblastoma (caregiver sample). Most survivors lived with their parents, not independently. Few survivors were employed (*n* = 2) and the majority were single (*n* = 10).

**Table 2:** Survivor characteristics

	Sex	Current Age	Employment status	Living status	Relationship status	Age at diagnosis	Diagnosis	Education
S1	F	16	Student	Family	Single	1	Pilomyxoid astrocytoma	Mainstream school
S2	M	30	Unemployed — but looking for work	Family	Single	6	Pilocytic astrocytoma	Mainstream school
S3	M	22	Unable to work due to illness or disability	Family	Single	10	Primitive neuroectodermal tumour	Mainstream school
S4	F	28	Working F/T ^a^	Partner	In a relationship	10	Pilocytic astrocytoma	Mainstream school
S5	M	18	Student	Family	Single	9	Oligodendroglioma	Mainstream school
S6	F	27	Working F/T ^a^	Family	Single	5	Pilocytic astrocytoma	Mainstream school
S7	M	24	Unemployed –but looking for work	Family	Single	10	Medulloblastoma	Mainstream school
S8	F	25	Unemployed –but looking for work	Family	Single	7	Medulloblastoma	Mainstream school
S9	F	26	Unable to work due to illness or disability	Family	In a relationship	2	Medulloblastoma	Mainstream school
S10	M	30	Unable to work due to illness or disability	Family	Single	5	Primitive neuroectodermal tumour	Mainstream school
S11	F	17	Student	Family	Single	4	Anaplastic ependymoma	Mainstream school

^a^F/T = full-time employment. P/T = part-time employment

**Table 3:** Caregiver characteristics

	Sex	Age	Employment status	Relationship status	Survivor sex	Age at diagnosis	Relationship to survivor	Survivor current age	Survivor diagnosis	Survivor education
C1	F	47	Working F/T	Married	F	1	Mother	16	Pilomyxoid astrocytoma	Mainstream school
C2	M	58	Working F/T	Married	M	6	Father	30	Pilocytic astrocytoma	Mainstream school
C3	F	55	Working P/T	Married	M	6	Mother	30	Pilocytic astrocytoma	Mainstream school
C4	F	56	Working F/T	Married	M	10	Mother	22	Primitive neuroectodermal tumour	Mainstream school
C5	F	50	Working F/T	Married	M	9	Mother	18	Oligodendroglioma	Mainstream school
C6	F	53	Working P/T	Married	M	10	Mother	24	Medulloblastoma	Mainstream school
C7	F	61	Working F/T	Divorced	F	7	Mother	25	Medulloblastoma	Mainstream school
C8	F	54	Caring for family/home	Married	F	2	Mother	26	Medulloblastoma	Mainstream school
C9	F	50	Working P/T	Married	M	5	Mother	30	Primitive Neuroectodermal tumour	Mainstream school
C10	F	49	Working P/T	Separated	F	4	Mother	17	Anaplastic ependymoma	Mainstream school
C11	F	40	Caring for family/home	Single	F	4	Mother	14	Medulloblastoma	Special educational needs school

We found four over-arching themes: (1) preferences for support and support services (unmet needs); (2) decline in support; (3) reasons for not obtaining adequate support; and (4) the role of long-term follow-up care. Key learning points are summarised in Table 4.

**Table 4:** Key learning points from each theme

Theme	Key learning point	Survivors and/or caregivers
1: Preferences for support and support services (unmet needs)	A preference for support that focuses on “achieving life events” such as living independently and making and maintaining relationships	Survivors
	A preference for support that assists with financial and practical elements of independent living. This may assist in helping both survivors and caregivers achieve those future goals	Caregivers
2: Decline in support	A drop in availability of services once the survivor completed treatment is a concern, especially as survivors become more aware of their health and the limitations they may face. Support services should endeavour to continue supporting survivors and their caregivers in long-term survivorship	Survivors and Caregivers
3: Reasons for not obtaining adequate support	Providing lay-friendly information is crucial for survivors so that they can understand the implications of their diagnosis/treatment	Survivors
	Resources/training for clinicians to facilitate the transition from addressing parents to addressing patients may be beneficial to follow-up care in general and support in particular	Survivors
	The dependence on informal support by caregivers highlights the variation in support services available due to location, funding, and unclear information. Consistency in support services is key to ensuring caregivers receive adequate professional support	Caregivers
4: The role of long-term follow-up care	The need for continuity of support. The continuation of long-term follow-up was “easier” when the clinical teams remained the same. Childhood cancer survivors benefit from joint working practices especially during the transition from child to adult services	Survivors and caregivers

### Theme 1: preferences for support and services

#### Achieving key life events

For caregivers and survivors, achieving “normal” life goals such as paid employment is very important, and the prospect of not achieving these caused concern. Most survivors experienced difficulty or were unable to find employment. Survivors expressed that they need more information on gaining employment. Unmet support needs included help with finding employers who are “disability friendly”, help preparing CVs/application forms, and interview support. Survivors’ financial issues were a major concern to caregivers, in particular if and how the survivor could support themselves.

The main challenges, well is finance, financial…It’s stressful cos I want [survivor name] to have some income, you know, cos I can’t support her, do you know what I mean…and that’s what worries me. [C8, F, 58^1^]

Difficulties with employment and finances are linked with independent living. Almost all survivors were still living with their family. They hoped to someday live independently but acknowledged this may take time.

I don’t even know how to cook now and I’m 27 and that’s because it’s very slow…I’m still picking up the pieces now…And how long I’m going to be picking them up for I don’t know? [S6, F, 27]

While independent living was a key goal for survivors, caregivers highlighted that for some (e.g. those with many physical impairments or learning difficulties prompting a need for continued intensive support) this goal is not achievable. Caregivers’ major concern was what would happen when they were no longer able to care for them.

That’s one of my biggest fears, not saying [name of wife] couldn’t look after him but…erm if owt happened to me then how would they cope? [C2, M, 58]

Caregivers expressed a need for more information on support to help survivors transition into independent or assisted living. This would help them plan ahead and decrease their anxiety about the future.

#### Developing a social network

Survivors and caregivers discussed that forming social relationships has been difficult. Some survivors indicated they would like support with making and maintaining friendships, finding social interactions difficult; “Like sometimes in my head I’m thinking ‘don’t trip up, don’t trip up’” *[S1, F, 16]*. Several survivors said that they would like to meet other survivors, but connecting with peers (i.e. similar age group) is the most important factor. Caregivers were equally worried about their child’s lack of social life and were supportive of meeting individuals with shared experiences through organised social support groups.

They’ve all got something in common, they don’t have to be talking about that all the time but they can all find this common ground. [C8, F, 54]

#### Navigating the future

Caregivers recalled that during the survivor’s diagnosis and treatment, they were not always aware of potential late effects, or they may not have fully realised at the time.

I’ve kind of had to go back over it myself to make it make sense again now, cos I think you’re given a lot of information in the early days but it just doesn’t go in… [C10, F, 49]

Caregivers highlighted that better timing of information on late effects and the need for ongoing contact and support alongside the survivors changing needs were vital. As this timing is difficult to get right, they suggested that such information could be reintroduced regularly.

#### Personalised, individualised mental health support

Mental health services were considered crucial. Survivors explained this was still needed long-term due to a growing understanding of their diagnosis and the experience of late effects.

I had counselling when I was 7 but I still want help now I’m 25... I don’t want that help to have just stopped…cos there’s still things that are happening and changing… the side effects never leave you if you know what I mean? [S8, F, 25]

The majority of survivors indicated that although they had received counselling during treatment, this subsided in later life. Some survivors stated that they would appreciate having a mentor or somebody who could provide 1-1 help. This was echoed by caregivers, some of whom felt that this was one of the greatest unmet needs.

I would like there to be like a mentor or a person who you could ring…somebody that you could contact to talk to about that certain thing that you want information on. [C8, F, 54]

Survivors wanted this mentor to have knowledge of brain tumours to help guide them through life experiences complicated by late effects. Caregivers wanted a reliable point of contact.

### Theme 2: decline in support

#### Life after education

Survivors and caregivers described support from education generally as positive. Support provided included care assistants (e.g. for mobility/personal care), additional time, or adapted educational aids.

I had modified papers, they were on green paper and a bigger font…It’s the spacing really that’s more helpful. [S11, F, 17]

High schools and colleges were often given formal advice and guidance to help them support survivors (e.g. through vision support officers). Survivors and caregivers described that difficulties started after finishing education, when support stopped and survivors felt at a loss of what to do next.

When I finished college it’s like – what am I supposed to do after that? [S10, M, 30]

#### Diminishing support getting further away from treatment

All caregivers were particularly positive about support received from clinical teams, charities, and support services, while their child was in treatment or acute care. However, many felt that support fell away as the child moved further away from treatment.

At the time it was just hospitals all the time. And I think [survivor] felt quite safe and I think it sounds quite a strange thing to say but we both felt quite safe but when you come out of hospital…you feel really lost… [C4, F, 56]

Caregivers and survivors described being unable to access support services once they reached adulthood.

### Theme 3: barriers to not obtaining adequate support

#### Practical barriers to accessing support

Caregivers said they were not aware of long-term support available or how to access support. “We didn’t really know what other support groups were in place…so we didn’t really know where you would go and look” [C1, F, 47]. Other barriers include support or information not being in an accessible format for survivors (e.g. due to vision/cognitive issues), long waiting lists, and lack of funding.

The waiting lists for everything are just immense…one thing we’ve been trying to get help with is to see a psychologist…we’ve still not seen a psychologist and this is 2 years on [C9, F, 50]

Equally, location of support services was challenging, complicated by difficulties with transportation. This was especially salient in participants living in rural areas:

A lot of the problem we have is because were in this dead zone – were surrounded by big Cities, it means we have to travel to the City to do anything [C9, F,50]

Having negative experiences of support services could cause reluctance to access support. Some describe bad experiences which include being let down or forgotten about.

They [the charity] sent a lady who was going to take him out and meet up with some other young man quite near us but that never transpired. [C6, F, 53]

Another issue identified is the lack of brain tumour specific support. Some survivors received support from cancer charities but felt that this was not suitable for their experiences, in particular their late effects.

I seeked out help but I found they always put me in learning disability groups but not brain tumour type… [S7, M, 24]

#### “Getting on with it”: emotional barriers to support

Survivors and caregivers explained a main reason for not having accessed support was that they were just “getting on” with life. Most caregivers had never received any formal support for themselves (e.g. counselling), instead relying heavily on friends or family for emotional support. Caregivers appeared to not prioritise their own needs, often saying they do not need it or choosing to cope on their own:

It’s hard but it’s called life and you have to get on with it! [C8, F, 54]

Survivors also expressed a positive outlook on life, and if they could, they chose not to seek formal support and manage on their own:

Like with the emotional stuff I just get on with it. There’s nothing like…like my chemo does, did affect me but I’ve just got over it. [S5, M, 18]

Both survivors and their caregivers expressed that “getting on with it” is synonymous with “seeming to be in control of the situation”. Survivors typically saw this as a positive outcome, but some caregivers indicated they had few choices but to “get on with it” and cope.

### Theme 4: the role of long-term follow-up care

#### Importance of follow-up care

Survivors and caregivers said the main benefit from attending long-term follow-up appointments was reassurance. Survivors described these appointments as a reassuring yearly check-up. Equally, caregivers found these appointments a valuable opportunity to ask questions and check late effect symptomology.

Just to get checked up, it’s an MOT [annual test of vehicle safety in the UK]. [S1, F, 16]

Many caregivers also said this service signposts them to other supports (e.g. charities) which could help the survivor live better.

#### Transition from children’s to adult services

Some survivors and caregivers described the transition from children’s to adult services as uncertain and confusing. Some survivors and caregivers said more support and information is needed to aid this transition.

The transition was easier where there was continuity in clinical staff and/or hospital.

It was quite an easy transition, it was same people, same place! It is that familiarity, it’s that person…I don’t have to explain everything, I don’t have to explain everything with [survivor], cos they know – they’ve been there all the way through! [C9, F, 50]

#### How follow-up care could be improved

A small number of survivors indicated that clinicians did not engage with them enough or fully explain their medication or treatments.

They need to engage more with the child than they do with the parents… I didn’t understand why I was on medication, apart from my Mum telling me…but a consultant physically did not tell me why I was on it, so when I would go pick my prescriptions up at the age of 18 and the consultants saying why are you on this…I would say ‘I don’t know’. [S6, F, 27]

With many survivors struggling with cognitive deficits, the use of lay language is essential. Survivors wanted clinicians to check their understanding:

Sometimes some of the doctors say things…again it’s a bit difficult to understand them or process what they’re saying, I don’t quite understand the terminology. [S8, F, 25]

Caregivers hoped support could be improved by extending it beyond just medical care to holistic or social well-being care, e.g. job application support.

Survivors and their caregivers report a number of similar goals with subtle differences in their unmet support needs; in particular they both express a desire for more emphasis and information on future outcomes. For survivors this includes the ability to live independently and assistance with attaining this goal. Caregivers expressed a need for financially centred information.

## Discussion

This qualitative study provides insight into the unmet supportive care needs of TYA childhood brain tumour survivors and their caregivers. Our findings suggest that survivors are greatly concerned with their ability to live independently, find employment, and build and maintain social connections. Previous survey-based TYA cancer survivor research (not brain tumour-specific) similarly highlighted the need for support with making and maintaining friendships, but did not yet cover “achieving life events” [32]. Parents are very aware of the need for socialisation outside of the family, a finding supported by previous literature [33]. We found that caregivers are concerned with what will happen if they can no longer support the survivor both practically (financially and physically) as well as emotionally. Previous TYA brain tumour research similarly reports caregiver concerns over inadequate financial support and a decline in support available after treatment [34, 35].

Adolescence and early adulthood is a unique and complex developmental phase consisting of sensitive physical and emotional challenges [36], complicated by survivors’ experiences of varying degrees of late effects. It is therefore unsurprising that survivors in our study most often wanted mental health and one-to-one support. Both survivors and caregivers explained that they felt a drop in service availability after treatment. This was of particular concern to survivors who, as they grew older, understood more about their health and late effects. Yet, mental health support is typically not part of usual follow-up care, highlighting an area of significant unmet needs. While this is in line with previous research suggesting that more comprehensive follow-up services for childhood cancer survivors are required [22–24, 37], our findings confirm these needs are still unmet in TYAs.

In our study, barriers to accessing formal support included funding and unclear or conflicting information which could create confusion about the applicability of support options. Location and transport were highlighted as barriers by caregivers living in rural areas in particular. Participants stressed the importance of providing lay-friendly information and providing it to survivors directly instead of via caregivers. This is supported by studies in childhood cancer survivors [33]. In addition, resources or training for clinicians to facilitate the transition from addressing parents to survivors may be beneficial to follow-up care in general and support in particular.

The need for continuity of support was also highlighted; participants explained long-term follow-up was “easier” when clinical staff or services remained the same. Survivors expressed a benefit from seeing “familiar faces”. Indeed, childhood cancer survivors can benefit from multimodal and joint working practices in transitioning from child to adult services [38]; this is particularly important for survivors who are not living independently [39].

While this study holds its strengths in talking to both survivors and caregivers using in-depth qualitative interviews, it does have its limitations. We recruited participants attending long-term follow-up clinics, thus likely underestimating unmet needs of survivors and caregivers who do not access these clinics. Moreover, we defined TYA as 13–30 years old, while throughout the oncology literature, there is great disparity over the definition of TYA age ranges [40]. While we aimed for maximum variation sampling, the final sample may not reflect non-English-speaking families, survivors aged under 16 and those diagnosed between 10 and 14. When younger survivors were approached for recruitment their parents often declined due to survivors’ cognitive deficits. A small number of non-English-speaking survivors and caregivers were not eligible for participation due to lack of translation resources. These may be subgroups with even higher unmet needs.

This study highlights survivors’ and caregivers’ unmet support needs, barriers to obtaining support, and improvements that could be made to long-term follow-up care. The practical suggestions made can provide stepping stones to improving long-term follow-up care, enhancing quality of life for TYA childhood brain tumour survivors and their caregivers.

## Data Availability

Data are held securely by the research team and may be available upon reasonable request and with relevant approvals in place.

## Footnotes

1 C = caregiver number. S = survivor number. F = female. M = male. Number = current age
